# Novel EGFP reporter cell and mouse models for sensitive imaging and quantification of exon skipping

**DOI:** 10.1038/s41598-020-67077-4

**Published:** 2020-06-22

**Authors:** Yuko Hara, Yoshitaka Mizobe, Yukiko U. Inoue, Yasumasa Hashimoto, Norio Motohashi, Yoshiaki Masaki, Kohji Seio, Shin’ichi Takeda, Tetsuya Nagata, Matthew J. A. Wood, Takayoshi Inoue, Yoshitsugu Aoki

**Affiliations:** 1 Department of Molecular Therapy, National Institute of Neuroscience, National Center of Neurology and Psychiatry, Tokyo, Japan; 2 Department of Biochemistry and Cellular Biology of Neuroscience, National Institute of Neuroscience, National Center of Neurology and Psychiatry, Tokyo, Japan; 3 Department of Life Science and Technology, Tokyo Institute of Technology, Kanagawa, Japan; 4 Department of Neurology and Neurological Science, Tokyo Medical and Dental University, Tokyo, Japan; 5 Department of Paediatrics, University of Oxford, South Parks Road, Oxford, United Kingdom

**Keywords:** Assay systems, Drug delivery

## Abstract

Duchenne muscular dystrophy (DMD) is a fatal X-linked disorder caused by nonsense or frameshift mutations in the *DMD* gene. Among various treatments available for DMD, antisense oligonucleotides (ASOs) mediated exon skipping is a promising therapeutic approach. For successful treatments, however, it is requisite to rigorously optimise oligonucleotide chemistries as well as chemical modifications of ASOs. To achieve this, here, we aim to develop a novel enhanced green fluorescence protein (EGFP)-based reporter assay system that allows us to perform efficient and high-throughput screenings for ASOs. We design a new expression vector with a CAG promoter to detect the EGFP fluorescence only when skipping of *mdx*-type exon 23 is induced by ASOs. Then, an accurate screening was successfully conducted in C57BL/6 primary myotubes using phosphorodiamidate morpholino oligomer or locked nucleic acids (LNA)/2′-OMe mixmers with different extent of LNA inclusion. We accordingly generated a novel transgenic mouse model with this EGFP expression vector (EGFP-*mdx*23 Tg). Finally, we confirmed that the EGFP-*mdx*23 Tg provided a highly sensitive platform to check the effectiveness as well as the biodistribution of ASOs for exon skipping therapy. Thus, the assay system provides a simple yet highly sensitive platform to optimise oligonucleotide chemistries as well as chemical modifications of ASOs.

## Introduction

Duchenne muscular dystrophy (DMD) is a highly common and fatal X-linked disorder caused by nonsense and frameshift mutations in the dystrophin (*DMD*) gene. No effective treatments are currently available for DMD; however, recently, several therapeutic approaches for DMD have been investigated^1–3^. Among them, exon skipping therapy is a highly advanced approach with great potential to effectively treat DMD. This approach uses antisense oligonucleotides (ASOs) to modulate the pre-mRNA splicing of *DMD* transcripts to restore the disrupted *DMD* reading frame. ASOs are designed to hybridise and mask the splicing signals of a target exon and thus prevent its inclusion in the final mRNA by the splicing machinery, thereby leading to the synthesis of a shorter, but partially functional protein^4–9^ (Supplementary Fig. S1a,b).

Eteplirsen, which targets *DMD* exon 51, was conditionally approved by the US Food and Drug Administration (FDA) as the first antisense drug for the treatment of DMD patients in September 2016. In addition, there are many candidates ASOs for exon skipping therapy, including those targeting other exons (https://clinicaltrials.gov/). To achieve efficient dystrophin protein restoration due to exon skipping, the selection of appropriate lead oligonucleotide chemistries and chemical modifications in addition to sequences of ASOs is vital^10–16^. Generally, ASO candidates are screened by injecting them into the mouse skeletal muscles, then collecting these tissue samples to evaluate the exon skipping by reverse transcription PCR (RT-PCR), and finally confirming the recovery of DMD expression by western blotting. However, these steps are often time-consuming and need considerable numbers of mice, which is not suitable for the rapid screening of multiple candidate drugs. Therefore, a simple and accurate primary screening method is required.

Here, we designed a new ASO drug-screening system with an enhanced green fluorescent protein (EGFP)-based reporter vector with a CMV early enhancer/chicken β actin (CAG) promoter. In the reporter vector, exon 23 of *Dmd* with nonsense mutation same as *mdx* mouse is inserted into a split EGFP (EGFP-exon23/pCAGGS) to detect the EGFP fluorescence, only when skipping of exon 23 with ASOs restores the EGFP-reading frame and expression of EGFP protein. By using the vector, we demonstrated that we could screen highly potent chemical modifications of ASOs based on the intensities of EGFP signal using a plate reader. With such an effective screening system, we revealed that we could precisely measure the exon skipping efficiency for candidate ASOs in a short time compared with the conventional method. We were also able to identify the optimal LNA/2′-OMe mixmers with different extent of phosphorothioate modification to induce targeted exon skipping using this reporter system. We finally generated novel transgenic mice with the EGFP-based reporter (EGFP-*mdx*23 Tg mice) to successfully detect the biodistribution of ASOs *in vivo*. These results indicated the value of our system for the immediate screening of multiple candidate exon skipping drugs for DMD.

## Results

### Evaluation of mouse *Dmd* exon 23 skipping events by ASOs using our novel EGFP-reporter system in C57BL/6 primary myotubes

**Figure 1:**
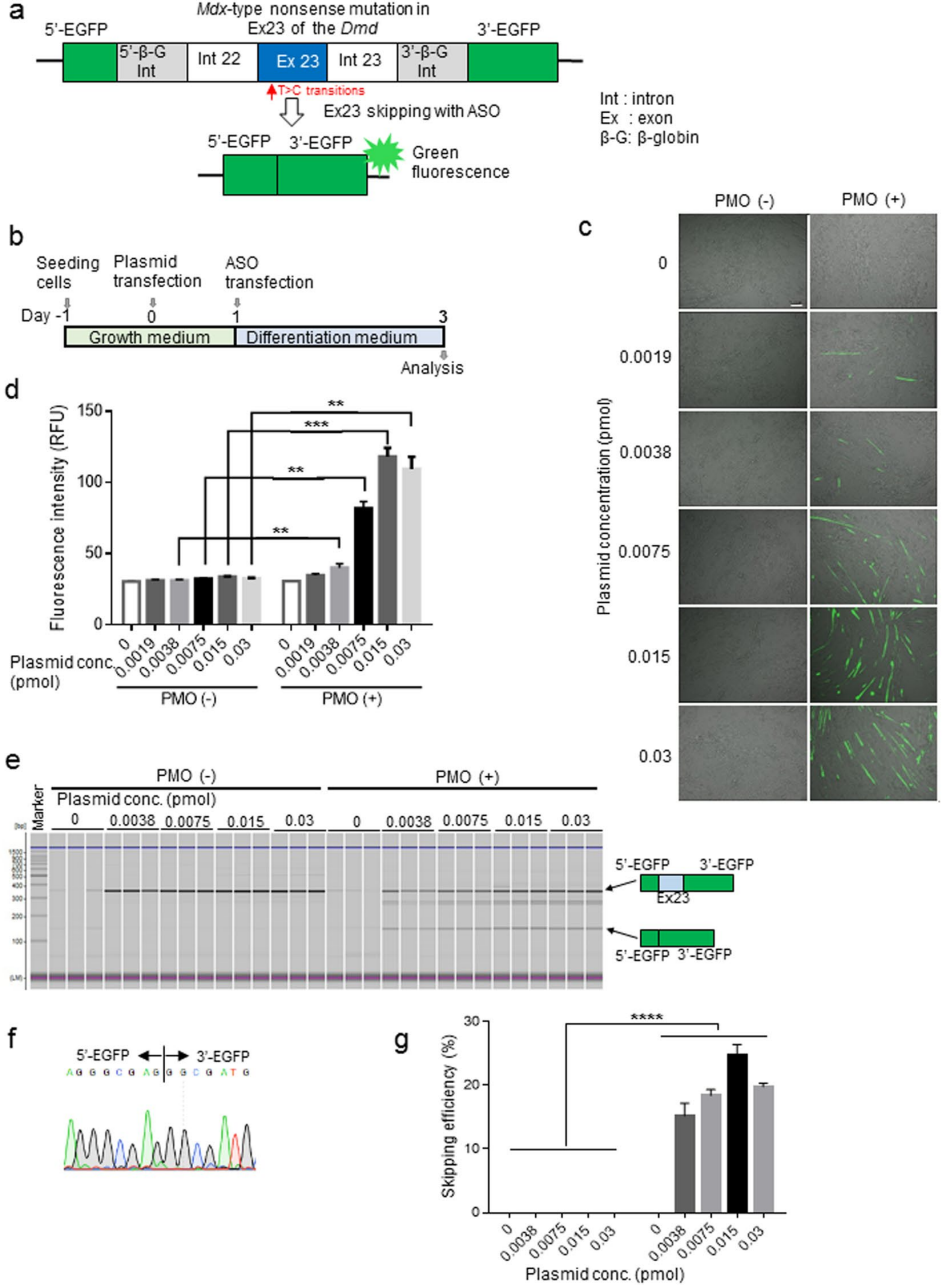
*In vitro* screening analysis using the *mdx*-exon23/enhanced green fluorescence protein (EGFP) vector. (**a**) The structure of the EGFP-exon 23 cassette to report dystrophin exon skipping is depicted: The EGFP gene is split into two regions, 5′-EGFP and 3′-EGFP, with the insertion of mouse dystrophin genomic region containing the exon 23 and neighbouring introns (Int 22 and Int 23). Skipping the exon 23 with antisense oligonucleotides (ASO) restores the EGFP open reading frame to express EGFP proteins. (**b**) Schematic diagram of *in vitro* experiments. (**c**) Representative images are showing the detection of EGFP expression by fluorescence microscopy. The cells in the left column are treated with increasing concentrations of the EGFP-exon23/pCAGGS vector plasmid only. The cells in the right column are treated with 10 μM phosphorodiamidate morpholino oligomer (PMO) and the indicated concentrations of the EGFP-exon23/pCAGGS vector. Note that EGFP expression is only detected in the presence of PMO. Scale bar, 100 μm. (**d**) Average fluorescence intensity of EGFP is measured using a plate reader (n = 5 replicates per group). Data represent the mean ± SEM. **P < 0.01 and ***P < 0.001. (**e**) RT-PCR analysis for exon 23 skipping is developed by a microchip-based capillary electrophoresis (MultiNA) system (n = 3 replicates per group). LM, lower marker dye; UM, upper marker dye. Noticeably, administration of PMO changes the transcripts from the vector. (**f**) Sequencing analysis of the 141-bp band detected by RT-PCR clarifies the exon skipping events by PMO. (**g**) Average exon 23 skipping efficiency is summarised by a bar graph (n = 3 replicates per group). Data represent the mean ± SEM. ****P < 0.0001.

We sought to develop a novel EGFP-*mdx*23/pCAGGS reporter vector to demonstrate the efficiency of specific ASOs for exon skipping. In this vector, the insertion of exon 23 from the *Dmd* gene with a *mdx*-type nonsense mutation together with its two flanking introns within the EGFP transcript, leads to the disruption of the EGFP reading frame. When specific ASOs skip exon 23, EGFP expression should be restored (Fig. 1a). We assumed that the levels of EGFP expression were correlated with the efficiency of exon skipping both *in vitro* and *in vivo*.

We then introduced the EGFP-*mdx*23/pCAGGS vector into wild-type (WT) C57BL/6 primary myotubes; after 24 h, we transfected the myotubes with the phosphorodiamidate morpholino oligomer (PMO), a modified form of ASOs, to target exon 23 of the *Dmd* gene. Two days after PMO transfections, we measured EGFP fluorescence in WT primary myotubes by fluorescence microscopy (Fig. 1b). We accordingly found that the number of EGFP-positive cells was positively correlated with the concentration of EGFP-*mdx*23/pCAGGS vector plasmid (Fig. 1c). Next, we measured the EGFP fluorescence intensity in the cells using a plate reader. We revealed that the fluorescence intensity was positively correlated with the vector plasmid concentration, and there were significant differences in intensity between PMO-treated and untreated WT primary myotubes (Fig. 1d). Interestingly, the cells transfected with 0.015 pmol PMO showed the most vigorous fluorescence intensity among the groups.

Subsequently, we performed RT-PCR to confirm the level of exon 23 skipping induced by PMO and analysed the efficiency of exon 23 skipping using a microchip electrophoresis system, MultiNA. As the results, we could obtain bands with a length of 141 bp indicating the occurrence of exon 23 skipping, and also found that no skipped bands were detected in the untreated WT primary myotubes (Fig. 1e). We performed sequence analysis of the 141 bp band, in which the *mdx*-exon 23 cassettes were expected to be spliced out, and confirmed that the EGFP fragment contained the inserted sequence (Fig. 1f). Thereafter, we found that the calculated efficiency of exon skipping enhanced with increasing plasmid concentrations. There were significant differences in exon skipping efficiency between the treated and the untreated myotubes.

Furthermore, the cells transfected with 0.015 pmol plasmids showed the highest exon skipping efficiency (Fig. 1g). These values were proportional to the fluorescence intensity of EGFP. EGFP fluorescence intensity, as measured using a plate reader, was found to be positively correlated with exon skipping evaluated by RT-PCR.

### Screening results from LNA and 2′-OMe mixmers that target mouse *Dmd* exon 23 in C57BL/6 primary myotubes

**Figure 2:**
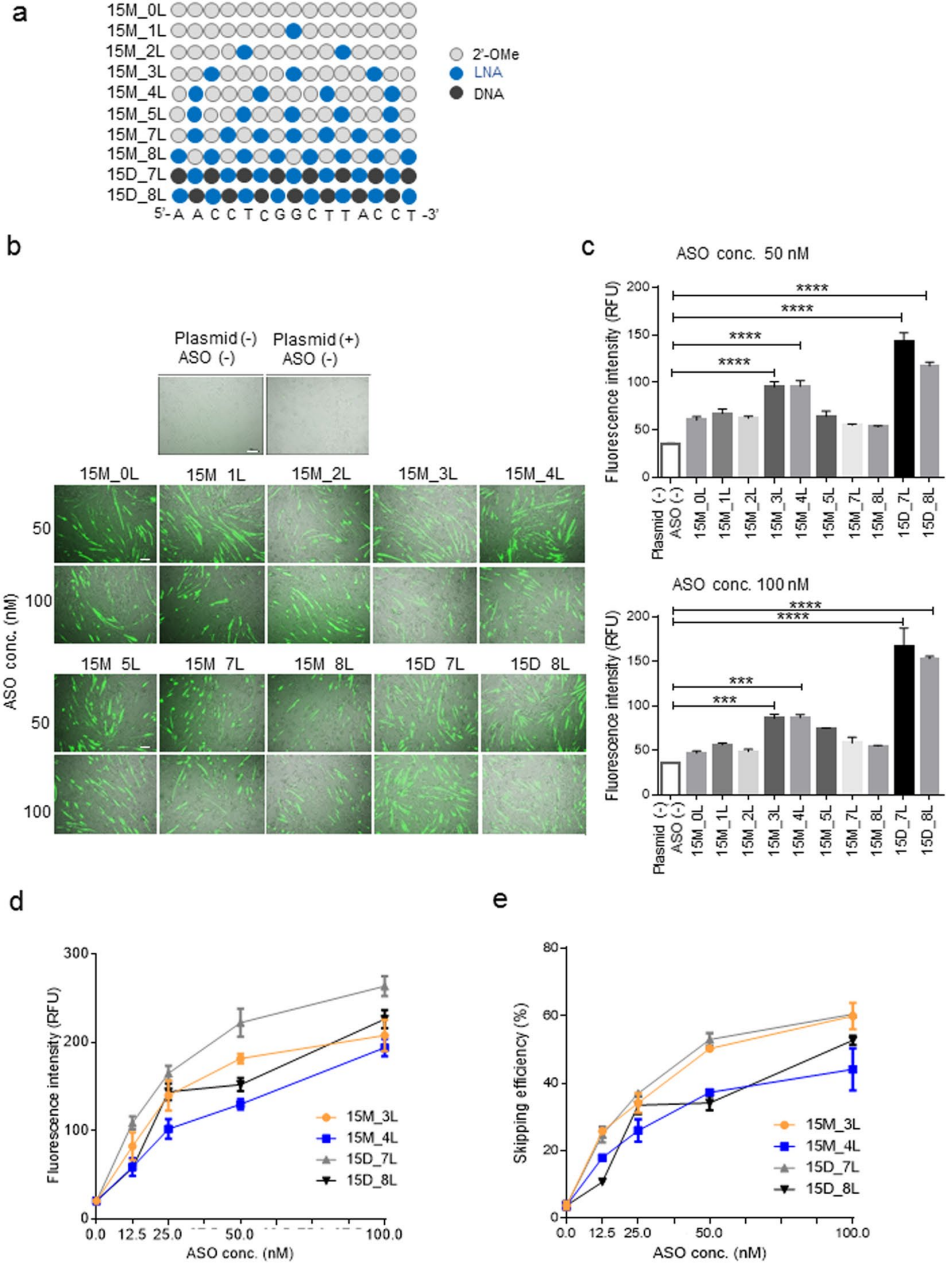
*In vitro* analysis using model antisense oligonucleotide (ASO) mixmers. (**a**) Schematic representation of the positions of locked nucleic acid (LNA) and 2′-OMe in the 15-mer oligonucleotide used in this screening. Each circle represents one nucleotide; the grey circle indicates 2′-OMe, and the blue and black circles indicate LNA and DNA, respectively. (**b**) Fluorescence microscopy images of wild-type (WT) primary myoblasts treated with LNA and 2′-OMe or LNA and DNA mixmers at 50 nM or 100 nM. Scale bar, 100 μm. (**c**) Average fluorescence intensity of EGFP in untreated or treated cells with mixmer is measured using a plate reader and indicated by bar graphs (n = 3 replicates per group). Data represent the mean ± SEM. ***P < 0.001 and ****P < 0.0001. (**d**) Average fluorescence intensity of EGFP in cells treated, as described below, is measured using a plate reader. Two lead mixmers, 15M_3L and 15M_4L, and two positive controls, 15D_7L and 15D_8L, are added at the indicated concentrations (0, 12.5, 50, 100 nM) (n = 5 replicates per group). Data represent the mean ± SEM. (**e**) Average exon 23 skipping efficiency of each mixmer is indicated by a line graph (n = 3 replicates per group). Data represent the mean ± SEM.

To confirm the usefulness of this reporter system, we designed eight novel LNA/2′-OMe mixmer ASOs (each mixmer includes 0 to 8 LNAs), as well as two LNA/DNA mixmers as positive controls (7 or 8 LNA)^17^ (Fig. 2a, Supplementary Fig. S2a–c). These mixmers had the same nucleotide sequences (5′-AACCTCGGCTTACCT-3′) with different extent of phosphorothioate modifications. We transfected each of the ten mixmers into C57BL/6 (WT) primary myotubes at a final concentration of 50 nM or 100 nM.

Two days after the transfection, we successfully detected EGFP fluorescence in mixmer-treated cells by fluorescence microscopy. On the other hand, we did not detect any EGFP fluorescence in cells without plasmid treatment (Fig. 2b). To assess the extent of mixmer-induced exon skipping, we measured the EGFP fluorescence intensity using a plate reader. The 15M_3L and 15M_4L mixmers showed significantly higher fluorescence intensity compared with the other mixmers (Fig. 2c). As shown in Fig. 2a, the 15M_3L mixmer contained three LNAs, while 15M_4L contained four LNAs. This result suggests that exon skipping efficiency was not correlated to the number of LNAs. It was surprising that the 15M_3L mixmer contained three LNAs induced significantly higher exon skipping efficiency than other mixmers contained five to eight LNAs.

Next, we examined the efficiency of the 15M_3L and 15M_4L mixmers by reducing the concentration of each mixmer to investigate the sensitivity of our assay system. WT primary myotubes were treated with 0 nM, 12.5 nM, 25 nM, 50 nM, or 100 nM of each mixmer; after 48 h, EGFP fluorescence intensity was evaluated by a plate reader (Fig. 2d, Supplementary Fig. S3a). The fluorescent intensity was consistent with the observation of fluorescence microscopy. Finally, we prepared total RNA samples from mixmer-transfected cells to determine the extent of exon 23 skipping in mRNA. The RT-PCR results showed that 15M_3L and 15M_4L induced exon skipping in a dose-dependent manner (Fig. 2e, Supplementary Fig. S3b).

### Screening results from mixmer-based ASOs in EGFP-*mdx*23 Tg primary myotubes

**Figure 3:**
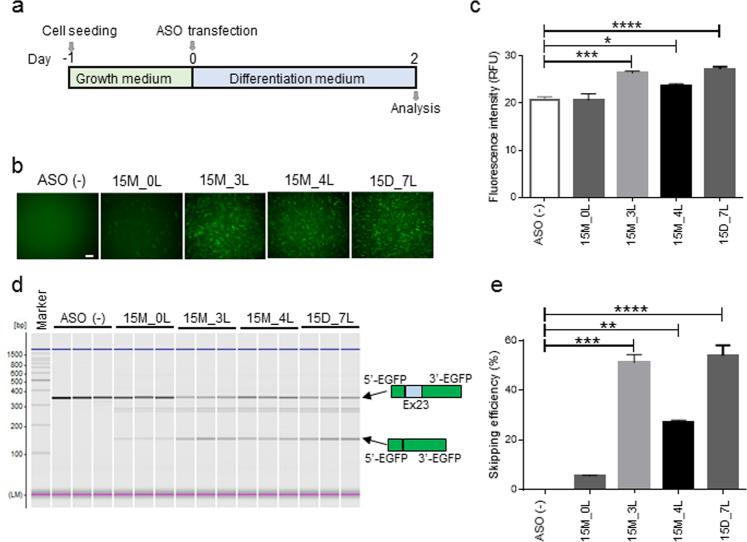
Transfection of LNA and 2′-OMe mixmers into isolated primary myotubes of EGFP-transgenic (Tg) mice. (**a**) Schematic diagram of *in vitro* experimental protocol using EGFP-*mdx*23 Tg primary myotubes. (**b**) Fluorescence images of Tg primary myotubes treated with 50 nM of 15M_0L, 15M_3L, 15M_4L and 15D_7L. (**c**) Average fluorescence intensity of EGFP is measured using a plate reader with 50 nM of the mixmers indicated (n = 6 per group). Data represent the mean ± SEM. *P < 0.05, ***P < 0.001, and ****P < 0.0001. (**d**) RT-PCR analysis for exon 23 skipping is developed by MultiNA. LM, lower marker dye; UM, upper marker dye. (**e**) Average band concentration of exon 23 skipping is indicated by a bar graph (n = 3 per group). Data represent the mean ± SEM. Antisense oligonucleotides: ASO.

We then generated the EGFP-mdx23 Tg mice to examine the exon skipping efficiency of ASOs *in vivo*. We obtained five founder lines for EGFP-*mdx*23 Tg mice. After confirming the stable germline transmission of the transgene, we determined its copy number. As the results, we found that the line 2 harbours the most adequate copy number for further experiments (Supplementary Fig. S4a,b). We thus isolated EGFP-*mdx*23 Tg primary myotubes from the line 2 mice, the copy number was 10 and examined the compositions of 15M_3L and 15M_4L, which showed the high skipping efficiency in WT primary myotubes. We simultaneously examined 15D_7L as an efficient ASO and 15M_0L as an inefficient ASO. We transfected each of the four mixmers at 50 nM into isolated EGFP-*mdx*23 Tg primary myotubes and two days after the transfection, and we measured EGFP fluorescence in the myotubes by fluorescence microscopy (Fig. 3a).

Consequently, high proportions of EGFP-positive cells were observed after transfection with the 15M_3L or 15M_4L mixmers, similar to the intensity observed in the positive control (15D_7L)-treated group. In addition, we reveal that a low proportion of EGFP-positive cells after transfection with 15M_0L (Fig. 3b). To evaluate the efficiency of mixmer-induced exon skipping of 15M_3L or 15M_4L, we measured EGFP fluorescence intensity using a plate reader (Fig. 3c). As the results, we found that the EGFP fluorescence intensity of the cells added with 15M_3L or 15M_4L was significantly higher than that of not added with mixmer. Finally, we performed RT-PCR to confirm the efficiency of exon 23 skipping using the EGFP-primer pairs. We detected exon 23-skipped bands (141 bp) in both 15M_3L- and 15M_4L-treated cells (Fig. 3d), and the percentage of exon skipping for both mixmers was higher than that for 15M_0L (Fig. 3e). This result well correlates with our results of mixmer transfection in WT myotubes.

### Investigation of exon skipping *in vivo* using EGFP-*mdx*23 Tg mice

**Figure 4:**
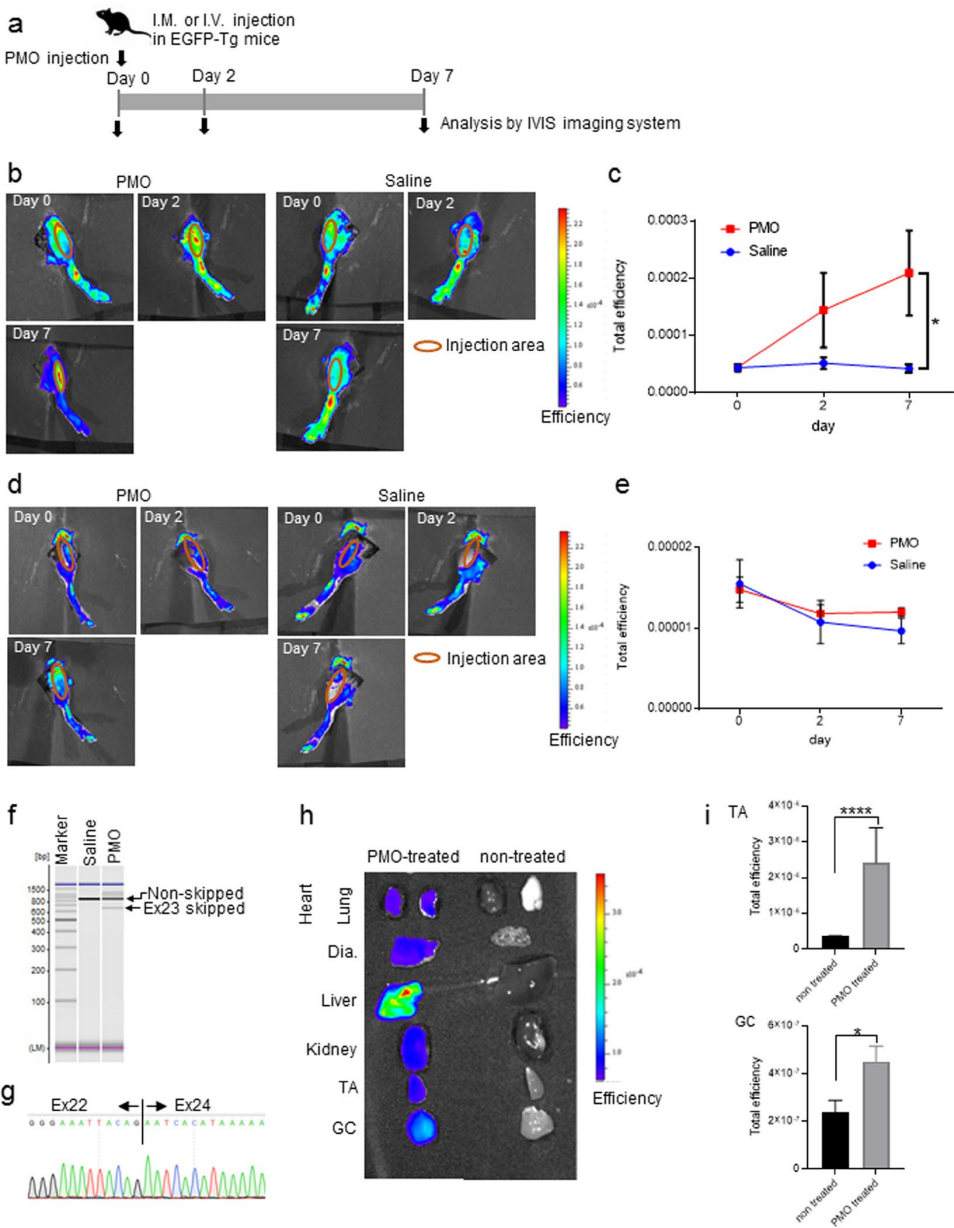
*In vivo* analysis of exon 23 skipping using EGFP-*mdx*23 Tg mice. (**a**) Schematic diagram of intramuscular injection of PMO. PMO is injected at 20 μM into the left TA of EGFP-*mdx*23 Tg or WT mouse, and saline is injected into the right TA as a negative control. Intramuscular EGFP fluorescence is analysed at day 0, 2, and 7 by the *In Vivo* Imaging System imaging (IVIS). (**b**) Analysis of intramuscular injection of PMO into EGFP-*mdx*23 Tg mice by IVIS imaging. Representative images are shown. (**c**) The total efficiency of EGFP-*mdx*23 Tg mice in the injection area. EGFP fluorescence intensity increases over time following the PMO injection. No significant changes are observed in the saline injection area (n = 3 per group). Data represent the mean ± SEM. *P < 0.05. (**d**) Analysis of intramuscular injection of PMO into WT mice by IVIS imaging as the negative control. Representative images are shown. (**e**) The total efficiency of WT mice in the injection area. No EGFP fluorescence is detected in the injection area (n = 3 per group). Data represent the mean ± SEM. *P < 0.05. Representative images are shown. (**f**) RT-PCR analysis of TA muscle tissue samples with or without PMO treatment. (**g**) Results of sequence analysis of the exon 23 skipped band detected by RT-PCR. (**h**) Representative images of *Ex vivo* analysis using IVIS images of systemic injection of PMO to EGFP-*mdx*23 Tg mice. Mice are injected with 640 mg/kg body weight PMO. The left column shows the muscles and organs of PMO-treated mice, and the right column shows those of mice treated with saline. (**i**) The total efficiency of EGFP expression in TA and GC muscles after systemic injection (n = 3 per group). Data represent the mean ± SEM. *P < 0.05, ****P < 0.0001.

We next used the EGFP-*mdx*23 Tg mice to assess the efficiency of exon skipping *in vivo*. To examine whether exon skipping could be detected in EGFP-*mdx*23 Tg reporter mice, we first intramuscularly injected PMO (20 μg) into the left TA muscles and saline into the right TA muscles as a control. We then evaluated exon skipping by using the *In Vivo* Imaging System (IVIS) on day 0, 2, and 7 (Fig. 4a). As a consequence, EGFP fluorescence in the PMO injection area increased in left TA muscles, while no changes were observed in the right TA muscles (Fig. 4b). We also observed significant differences in fluorescence intensity between tissues with and without PMO treatment at day 7 (Fig. 4c). As a control, we injected PMO or saline into WT mouse TA muscles; however, we found that the fluorescence signals were feeble in WT mice (Fig. 4d,e). To confirm that the induction of exon skipping occurred in TA muscles, we extracted RNA from the TA muscles and performed RT-PCR with primers flanking exons 22 and 25 of the *Dmd* gene (Fig. 4f), and successfully detected exon 23-skipped bands (648 bp) in PMO-treated TA muscles. We additionally performed RT-PCR using the extracted RNA to confirm the efficiency of exon 23 skipping using the EGFP-primer pairs and confirmed that the EGFP fragment contained the inserted sequence (Supplementary Fig. S5a,b).

Conversely, we detected only non-skipped bands (839 bp) in the saline-injected TA muscles. In addition, we confirmed via sequence analysis that the 648-bp band was missing exon 23 (Fig. 4g). Thus, we concluded that the levels of EGFP expression reliably reflected the efficiency of ASO *in vivo*.

Based on these results, we next performed a systemic injection of 640 mg/kg body weight of PMO to examine the induction of exon skipping using EGFP-*mdx*23 Tg mice. At 7 days after the systemic injection, we assessed exon skipping efficiency based on EGFP fluorescence in the heart, lung, diaphragm, liver, kidney, TA muscles, and GC muscles using the IVIS. Because EGFP has a detection wavelength close to that of erythrocytes, *ex vivo* analysis allowed us to evaluate EGFP fluorescence in each organ more accurately. EGFP fluorescence was virtually detected in the diaphragm, liver, kidney, TA, and GC of PMO-treated EGFP-*mdx*23 Tg mice, while almost no fluorescence was detected in the heart or lung (Fig. 4h). We additionally evaluated EGFP fluorescence in each organ and identified significant differences in fluorescence intensity between TA and GC muscles with and without PMO treatment (Fig. 4i).

## Discussion

Splice switching therapy using ASOs is one of the most promising approaches to treat DMD. By blocking abnormal splice sites in the targeted exon, ASOs are able to correct an abnormal splicing pattern of the gene. Therefore, ASOs are used to induce the skipping of target *DMD* exon(s) to restore the correct reading frame. The identification of effective ASOs capable of inducing exon skipping is a significant focus of research.

In this study, we developed a new EGFP-based reporter assay system to screen ASOs for DMD therapy. The *mdx* mouse, which has a nonsense mutation (C > T) in exon 23 of the *Dmd* gene, is a well-known DMD model. We targeted the exon 23 of the *Dmd* gene, which contains an *mdx*-type nonsense mutation (C > T) in exon 23, with our EGFP-based reporter to visualise and evaluate the efficiency of exon skipping.

EGFP is a bright, monomeric green fluorescent protein that is easily observable under a fluorescence microscope *in vitro*. An EGFP reporter has been used injection study in mouse models^18^, however, it is not ideal for *in vivo* observations because of the increase in background noise due to its detection wavelength being similar to that of red blood cells and melanocytes. To overcome this limitation, we incorporated a CAG promoter into the vector, which is a ubiquitous and robust promoter from the chicken *Ac* gene that is frequently used to induce high levels of gene expression in mammalian expression vectors^19–21^. In this way, we successfully detected fluorescence signals in primary myotubes using a plate reader and evaluated exon skipping efficiency in EGFP-*mdx*23 Tg mice using an *in vivo* imaging system.

To confirm the usefulness of this screening system, we first transfected PMO, which targets exon 23, into mouse primary myotubes, and detected EGFP fluorescence using a fluorescence microscope and fluorescence intensity using a plate reader. Our data suggested that this assay system produced similar results to those obtained using conventional methods such as RT-PCR. Therefore, it should be suitable to measure EGFP fluorescence intensity with a plate reader to evaluate exon skipping efficiency. This has the advantage of no necessity of detaching cells, extract RNA, and perform RT-PCR.

Next, we screened eight novel LNA/2′-OMe mixmer compounds as model compounds using this assay system. Our results revealed that two mixmers, which contained three and four LNA molecules, respectively, showed efficient exon skipping similar to that of the positive control. Because LNA has a high melting temperature, it is known to have high binding activity with RNA. On the other hand, it is known that LNA shows hepatic toxicity; thus, it is essential to minimise the number of LNAs in mixmer ASOs^22,23^. Therefore, if we can reduce the number of LNA molecules while keeping a high exon skipping efficiency, the risks of hepatic toxicity will be significantly reduced. Interestingly, we found that exon skipping efficiency did not improve as the number of LNAs increased in our assay system. According to our results, the exon skipping activity of the mixmers containing three or four LNA molecules in our 15-mer ASO showed the highest activity. Thus, our assay system could also effectively screen chemically modified ASOs rapidly *in vitro*. Similar results are reported that the LNA/DNA mixmer compounds have better efficacies than all-LNA ASO with an equivalent sequence for SMN2 exon inclusion in human spinal muscular atrophy patient fibroblasts (PMC5473822).

In exon skipping treatment, it is essential to know the dynamics of the ASOs *in vivo*. For this purpose, we generated EGFP-*mdx*23 Tg mice and performed quantitative PCR (qPCR) to estimate the transgene copy number. Among the five lines of EGFP-*mdx*23 Tg mice, line 4 showed the highest copy number. Based on our previous studies, Tg mice which have 8–10 copies are most useful. This is partly because high copy number founders tend to harbour multiple integration sites in the genome, making it challenging to maintain Tg lines stably. In this study, we have established primary myoblasts from different Tg lines to virtually find out that the Line 2 with a copy number 13 yields most stable results for the detection of EGFP due to exon 23 skipping *in vitro* and *in vivo* studies. Using Line 2 Tg mice, we investigated whether EGFP fluorescence could be detected with IVIS after the administration of PMO. EGFP fluorescence was detected after both intramuscular injections into TA muscles and systemic intravenous administration. In the case of intramuscular injection, EGFP fluorescence was strongly detected in the injection area. In the case of systemic administration, we detected EGFP fluorescence in the liver, kidney, GC muscles, and TA muscles. On the other hand, PMO was not delivered to the brain or heart. Because the CAG promoter is not directional to a specific organ, it is strongly expressed in organs in addition to skeletal muscle. In addition, EGFP fluorescence detected using the IVIS reflected the exon skipping-inducing effect on endogenous *Dmd*; thus, our EGFP-*mdx*23 Tg system is useful for systemic medical and kinetic evaluation of ASO efficacy. A limitation of the model is that it cannot be used to assess dystrophin restoration levels after ASO mediated exon skipping due to the nature of the model and, as a next step, we are currently developing EGFP-*mdx*23 Tg mice with *mdx* background.

In conclusion, we developed a CAGGS-EGFP-based reporter system capable of evaluating the exon skipping efficiency of ASOs. Our screening system and EGFP-*mdx*23 Tg mice provide a simple, fast and very sensitive approach to screen varieties of oligonucleotide chemistries as well as chemical modifications of ASOs, which could accelerate the development of highly efficient exon skipping drugs for DMD.

## Materials and Methods

### **EGFP-reporter vectors for*Dmd*exon 23 skipping**

The mouse *Dmd* EGFP-exon 23/pCAGGS reporter vector was constructed based on the procedure reported previously^24^. The EGFP sequence was split into two regions, 5′-GFP and 3′-GFP, through the insertion of a human β-globin intron sequence^9^ that also included the mouse *Dmd* exon 23 with nonsense mutation same as *mdx* mouse flanked by a 300-bp intronic sequence at each end (mouse intron 22 on the 5′-end and mouse intron 23 on the 3′-end). Then, the fragment was inserted into the pCAGGS vector by homologous recombination. The expression of EGFP was driven by the CAGGS promoter, which induces high gene expression throughout the body. Without ASO intervention, the vector expressed an out-of-frame EGFP/exon 23 chimeric transcript. Skipping exon 23 with ASOs restored the EGFP reading frame and the expression of EGFP protein.

### Animals

Eight- to twelve-week-old C57BL/6 male mice were purchased from Clea Japan Inc. (Tokyo, Japan). Four-week-old B6C3F1 females were obtained from Japan SLC (Tokyo, Japan). All animals were handled in accordance with the guidelines of the Experimental Animal Care and Use Committee of the National Center of Neurology and Psychiatry, Tokyo, Japan. The Experimental Animal Care and Use Committee of the National Institute of Neuroscience, National Center of Neurology and Psychiatry, Japan, approved all experimental protocols in this study.

### Generation of EGFP-reporter transgenic mice

B6C3F1 females were superovulated and mated to obtain fertilised eggs. EGFP-*mdx*23/pCAGGS transgenic mice (EGFP-*mdx*23 Tg mice) were generated by microinjecting the linearised EGFP-exon23/pCAGGS reporter vector into pronuclei of those fertilised mouse eggs to establish a transgenic founder cohort (F0). For all mouse experiments, six- to ten-week-old Tg/+ F2 mice with a copy number of 8–10 were used. The transgene copy number was estimated by quantitative PCR (qPCR) using the EGFP TaqMan probe #4400291 (Applied Biosystems, Foster City, CA, USA) (Supplementary Fig. S5)^25^. Briefly, qPCR on an ABI StepOnePlus real-time PCR system (ThermoFisher Scientific) was performed. Ten ng of genomic DNA samples or copy number standards with EGFP TaqMan probe were compared in triplicates to rigorously estimate the copy number from ΔCt values for standard curve samples.

### Synthesis of antisense oligonucleotides

The DmdE23D-25mer sequence (5′-GGCCAAACCTCGGCTTACCTGAAAT-3′), designed based on the boundary sequences of exon and intron 23 of the mouse *Dmd* gene, was used in this study. Phosphorodiamidate morpholino oligomer (PMO) was purchased from Gene Tools (Philomath, OR, USA). Locked nucleic acids (LNA)/DNA and LNA/2′-OMe mixmer-based ASOs with phosphorothioate backbone were purchased from Gene Design Inc. (Osaka, Japan).

### Primary myoblast preparation

For the preparation of primary myoblasts, anterior tibialis (TA) and gastrocnemius (GC) muscles were isolated from 10–12-week-old C57BL/6 and EGFP-*mdx*23 Tg mice^26^. Isolated TA and GC muscles were minced and incubated with 0.2% type II collagenase (Worthington Biochemical Corporation, Lakewood, NJ, USA)/Dulbecco’s modified Eagle’s medium (DMEM) for 30 min at 37 °C. Dissociated muscles were filtered through a 100-μm filter, followed by a 40-μm filter (Falcon, Corning Inc., Corning, NY, USA), and centrifuged at 200 × g for 5 min. The pellet was resuspended in growth medium and then plated on collagen-coated dishes (Falcon). During the first several passages of the primary cultures, myoblasts were enriched by preplating^27,28^.

### Cell culture

Isolated C57BL/6 primary myoblast cells were cultured in the growth medium, which consisted of DMEM (high-glucose, sodium pyruvate, and GlutaMAX supplement; Thermo Fisher Scientific, Waltham, MA, USA) supplemented with 20% fetal bovine serum (FBS; Thermo Fisher Scientific), 1% chicken embryo extract (CEE; US Biological, Salem, MA, USA), and 5 ng/ml basic Fibroblast Growth Factor (bFGF; Sigma Aldrich, St. Louis, MO, USA) at 37 °C with 5% CO_2_. The medium was changed every 2 days. Isolated EGFP-*mdx*23 Tg primary myoblasts were cultured in Ham’s F-10 Nutrient Mix (Thermo Fisher Scientific) supplemented with 20% FBS, 1% penicillin-streptomycin (PS; Thermo Fisher Scientific), and 5 ng/ml bFGF at 37 °C with 5% CO_2_. The medium was changed every 2 days. All plastic plates and dishes used for primary myoblast culture were coated with Matrigel-Growth Factor (Corning Inc.).

### Plasmid and ASO transfection

C57BL/6 primary myoblasts were seeded in 96-well black plates with clear bottoms (Corning 3603) at a density of 1 × 10^4^ cells per well and cultured in the growth medium. After 1 day, 0.0019, 0.0038, 0.0075, 0.015, or 0.03 pmol EGFP-exon23/pCAGGS reporter vector was transfected into the cells using Lipofectamine LTX and PLUS Reagent (Thermo Fisher Scientific) according to the manufacturer’s instructions. After 1 day, the culture medium was replaced with differentiation medium, which consisted of DMEM supplemented with 2% horse serum (Thermo Fisher Scientific); next, 10 μM PMO per well were transfected using Endo-porter (Gene Tools) according to the manufacturer’s instructions.

EGFP-*mdx*23 Tg primary myoblasts were seeded in 96-well black plates with transparent bottoms at a density of 1.5 × 10^4^ cells per well and cultured in the growth medium. After 1 day, the culture medium was replaced with differentiation medium, and PMO was transfected as described above.

For mixmer transfection, Lipofectamine 3000 (Thermo Fisher Scientific) was used according to the manufacturer’s instructions.

### Measurement of EGFP fluorescence intensity

Two days after the ASO treatment, the average EGFP fluorescence intensity per well was measured by a plate reader (Synergy HTX; BioTek, Winooski, VT, USA). The cells were visualised using the KEYENCE BZ-9000 fluorescence microscope (Keyence, Osaka, Japan).

### RNA isolation and reverse transcription PCR analysis

Total RNA was isolated from primary myotubes using the RNeasy Mini kit (Qiagen, Hilden, Germany) according to the manufacturer’s instructions. Using 100 ng RNA, single-strand cDNA was synthesised using the High Capacity cDNA Reverse Transcription Kit (Thermo Fisher Scientific) according to the manufacturer’s instructions. Then, reverse transcription PCR (RT-PCR) was performed using ExTaq HS (Takara Bio, Shiga, Japan). The primer sequences for RT-PCR were forward 5′-GACGTAAACGGCCACAAGTT-3′ and reverse 5′-ACCACCCTGACCTACGGC-3′. The PCR conditions were as follows: 32 cycles of 10 s at 98 °C, 30 s at 63 °C, and 90 s at 72 °C.

To extract total RNA from TA muscles, muscle tissues were homogenised using Bead Smash12 BS-12R (Waken B Tech Co, Ltd., Kyoto, Japan) and centrifuged at 2,500 rpm for 150 s at room temperature. Then, total RNA was extracted using the RNeasy Fibrous Tissue kit (Qiagen) according to the manufacturer’s instructions. Next, cDNA was synthesised as described above, and RT-PCR was performed using the forward primer 5′-ACCACCCTATCAGAGCCAAC-3′ and reverse primer 5′-CTGGCGGCATATGTGATCC-3′ to amplify of the mouse dystrophin exons 20–25 transcript. The PCR conditions were as follows: 35 cycles of 10 s at 98 °C, 30 s at 55 °C, and 30 s at 72 °C.

### *In vivo* assay using EGFP-*mdx*23 Tg mice

For intramuscular injection, 20 μg PMO was injected into the left TA muscles of EGFP-*mdx*23 Tg mice, and saline was injected into the right TA muscles as a control. PMO and saline were also injected into the TA muscles of C57BL/6 mice as negative controls. EGFP fluorescence was evaluated using the IVIS (PerkinElmer, Waltham, MA, USA) on day 0, 2, and 7.

On day 7, TA muscles were collected for RT-PCR, which was performed as described above. The PCR products were analysed using the microchip electrophoresis system MCE-202 (MultiNA; Shimadzu Corporation, Kyoto Japan). The efficiency of exon 23 skipping was calculated using the following formula: the moles of exon 23-skipped transcript/(the moles of non-skip + the moles of exon 23-skipped transcript) × 100 (%). For systemic administration, 640 mg/kg body weight PMO was injected into the retro-orbital venous sinus of EGFP-*mdx*23 Tg mice, and the same volume of saline was injected into negative control mice. Seven days after systemic injection, mice were sacrificed and dissected TA and GC muscles, brain, heart, lung, diaphragm, liver, and kidney. The EGFP fluorescence intensity in the organs was evaluated *ex vivo* using the IVIS.

### Statistical analysis

All data are presented as the mean ± standard error of the mean (SEM). Statistical analysis was performed using GraphPad Prism version 6.01 (GraphPad Software Inc., La Jolla, CA). Statistical significance was assessed by one-way analysis of variance (ANOVA) with Dunnett’s multiple comparisons test. A value of *P* < 0.05 was considered statistically significant.

## Supplementary information

Supplementary Figures S1-S5.

## Data Availability

All data generated or analysed during the present study are included in this published article (and its Supplementary Information Files).
