# Supplementary material for: Novel EGFP reporter cell and mouse models for sensitive imaging and quantification of exon skipping
Source: Sci Rep. 2020 Jun 22;10:10110. doi: 10.1038/s41598-020-67077-4 (PMC7308408; doi:10.1038/s41598-020-67077-4)
Supplement: Supplementary file 1 — Supplementary Figures S1-S5. [file 41598_2020_67077_MOESM1_ESM.pdf]

Supplementary information

Novel EGFP reporter cell and mouse models for sensitive imaging and quantification of exon skipping

Yuko Hara[1], Yoshitaka Mizobe[1], Yukiko U. Inoue[2], Yasumasa Hashimoto[1], Norio Motohashi[1], Yoshiaki Masaki[3], Kohji Seio[3], Shin'ichi Takeda[1], Tetsuya Nagata[4], Matthew JA Wood[5], Takayoshi Inoue[2], Yoshitsugu Aoki[1]*

[1] Department of Molecular Therapy, National Institute of Neuroscience, National Center of Neurology and Psychiatry, Tokyo, Japan.
[2] Department of Biochemistry and Cellular Biology of Neuroscience, National Center of Neurology and Psychiatry, Tokyo, Japan.
[3] Department of Life Science and Technology, Tokyo Institute of Technology, Kanagawa, Japan.
[4] Department of Neurology and Neurological Science, Tokyo Medical and Dental University, Tokyo, Japan.
[5] Department of Paediatrics, University of Oxford, South Parks Road, Oxford, United Kingdom.

- To whom correspondence should be addressed: Yoshitsugu Aoki.
- Tel: [+81-42- 346-1720]; Fax: [+81-42- 346-1750]
- Email: [tsugu56@ncnp.go.jp]

Figure S1. Schematic diagram of the exon-skip therapy for *mdx*-type mutation by ASO
Figure S2. Chemical structure of ASOs used in this study
Figure S3. Result from modified ASOs transfection to WT primary myotubes
Figure S4. Determining the copy number of EGFP-transgenic mice by qPCR
Figure S5. Successful exon 23 skipping evaluated by RT-PCR in PMO-treated tibialis anterior (TA) muscles of EGFP-mdx23 transgenic mice.

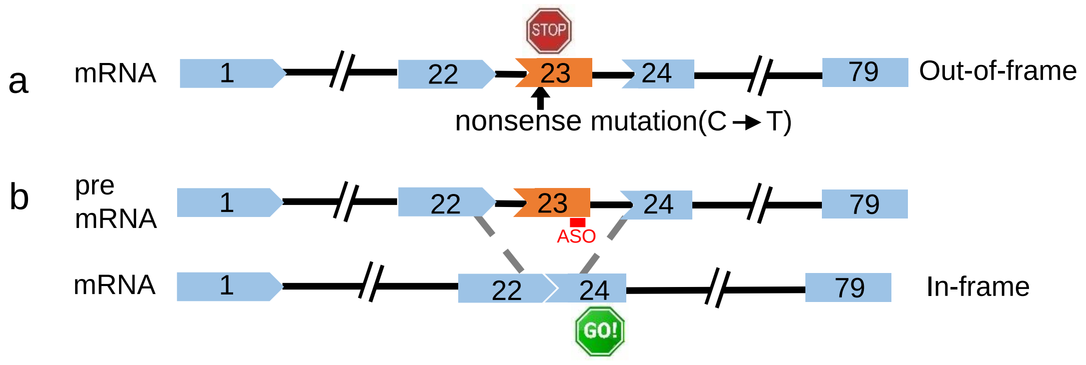

Figure S1. Schematic diagram of the exon-skip therapy for mdx-type mutation by ASO.
(a) Nonsense mutation of exon 23 (C > T) caused frameshift (out-of-frame) and produced a truncated dystrophin protein. (b) ASO targeted skipping exon 23 changes the out-of-frame transcript into in-frame one, allowing the synthesis of short but functional dystrophin. Antisense oligonucleotides: ASO.

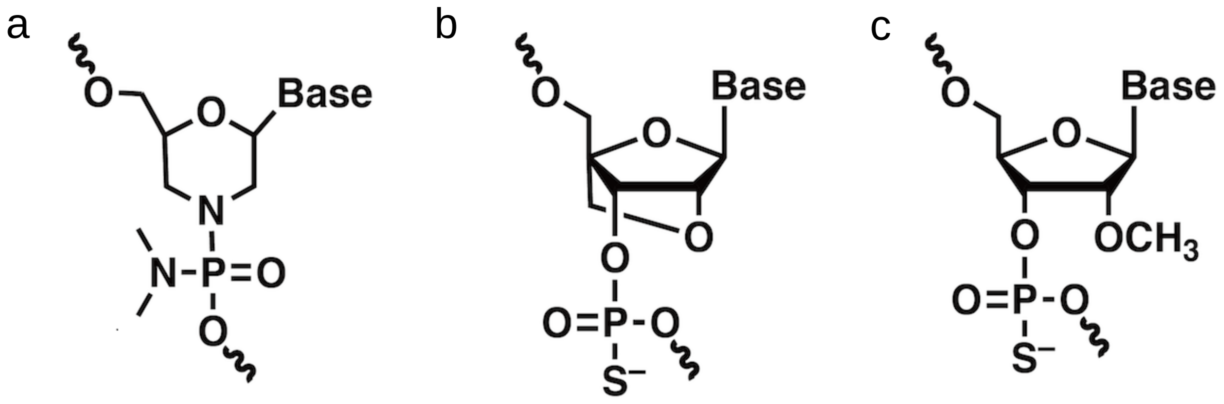

Figure S2. Chemical structure of antisense oligonucleotides using in this study.
(a) The backbone structure of phosphorodiamidate morpholino oligomer (PMO)
(b) The backbone structure of locked nucleic acid (LNA)
(c) The backbone structure of 2′-O-methyl

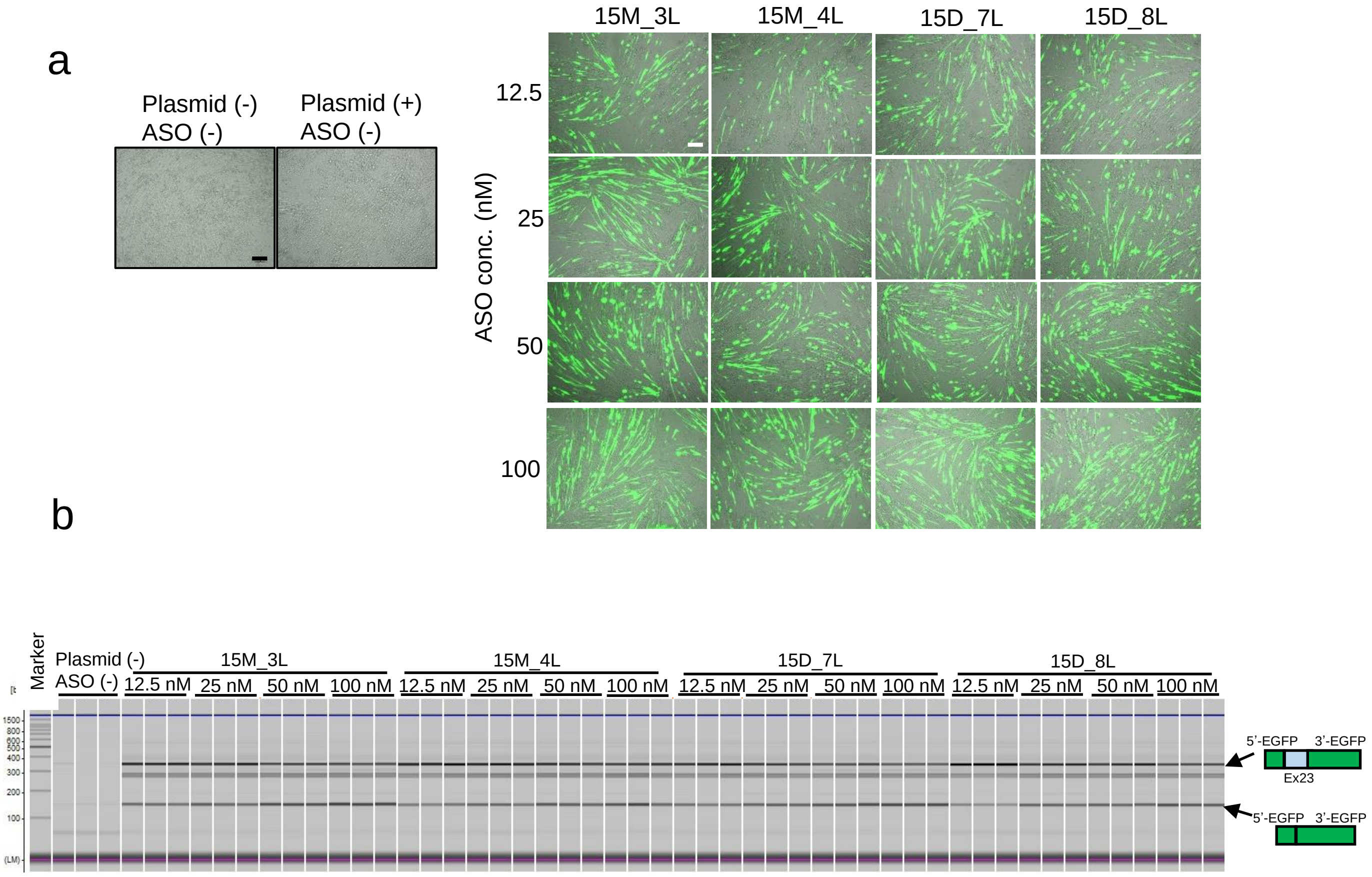

Figure S3. Transfection of LNA/2'-OMe mixmers in WT primary myotubes.

(a) Cells treated with various concentration of mixmers. Scale bar 100 µm.

(b) The result from RT-PCR for the detection of exon 23 skipping are outlined. The PCR conditions were as follows: 32 cycles of 10 s at 98℃, 30 s at 64 ℃, and 60 s at 72 ℃. Antisense oligonucleotides: ASO.

a

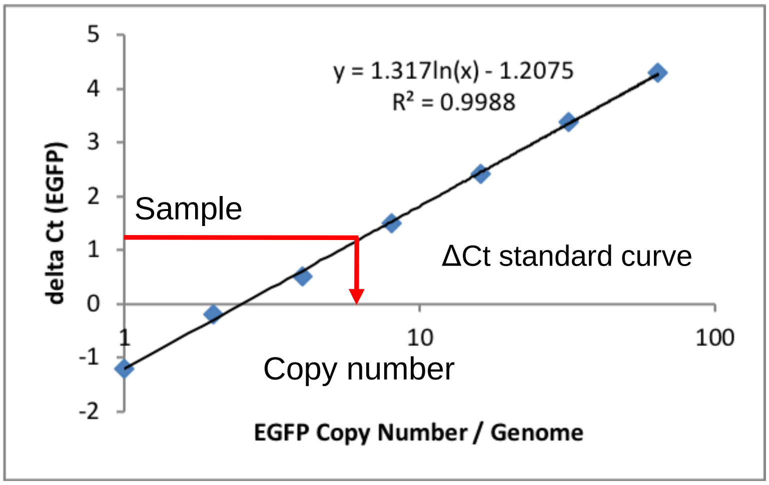

b

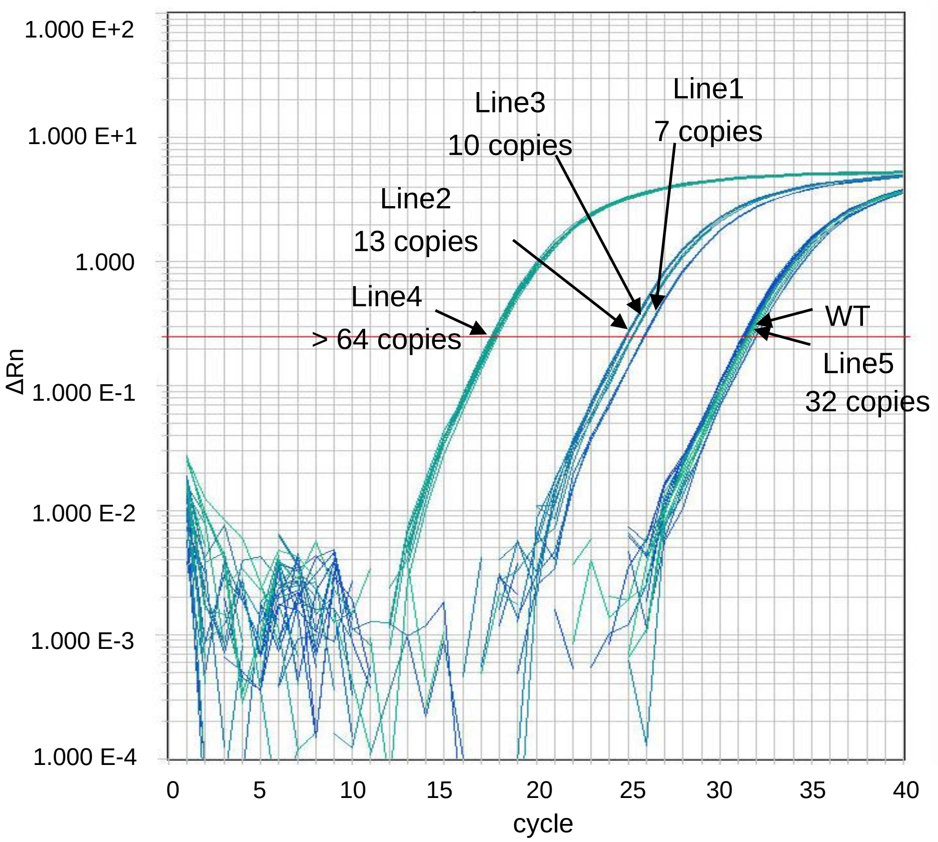

Figure S4. Determining the copy number of EGFP-mdx23 transgenic mice by qPCR

(a) A standard curve is drawn using EGFP-mdx23 / pCAGGS plasmid DNA.

(b) The copy number is calculated from the ΔCt value of the sample obtained by qPCR. Although Line 4 mice are estimated to carry more than 64 copies of the transgene, line 2 mice are mainly used for the study. This is because too many exogenous copies may cause instability of the integration sites as well as integrated gene cassettes over the crossing.

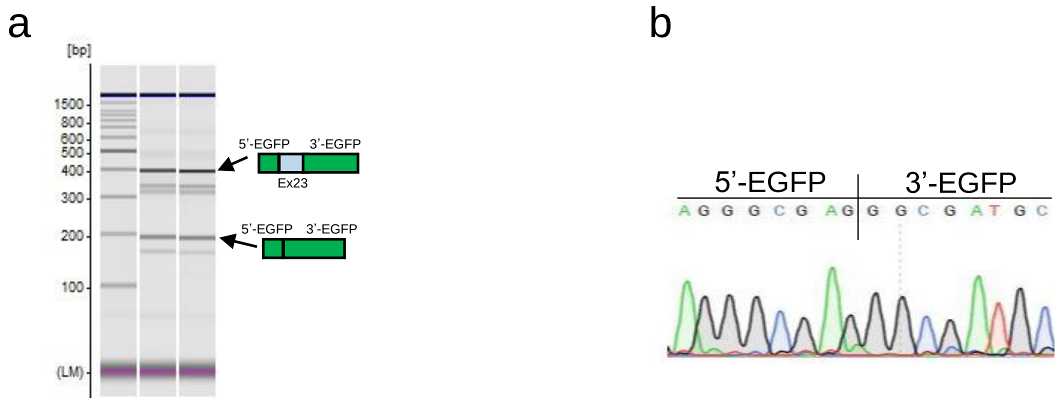

Figure S5. Successful exon 23 skipping evaluated by RT-PCR in PMO-treated tibialis anterior (TA) muscles of EGFP-mdx23 transgenic mice. (a) Result from RT-PCR for exon 23 skipping in TA muscles are summarised. (b) Confirmation of the sequence with exon 23 skipping. Note that 5 '- and 3'- fragments of EGFP are correctly fused.
